# Association between Placental Lesions, Cytokines and Angiogenic Factors in Pregnant Women with Preeclampsia

**DOI:** 10.1371/journal.pone.0157584

**Published:** 2016-06-17

**Authors:** Ingrid C. Weel, Rebecca N. Baergen, Mariana Romão-Veiga, Vera T. Borges, Vanessa R. Ribeiro, Steven S. Witkin, Camila Bannwart-Castro, Jose C. Peraçoli, Leandro De Oliveira, Maria T. Peraçoli

**Affiliations:** 1 Department of Gynaecology and Obstetrics, Botucatu Medical School, São Paulo State University, 18618–970, Botucatu, São Paulo, Brazil; 2 Department of Pathology and Laboratory Medicine, Weill Cornell Medical College – New York Presbyterian Hospital, New York, United States of America; 3 Department of Obstetrics and Gynecology, Weill Cornell Medical College, New York, United States of America; 4 Department of Microbiology and Immunology, Institute of Biosciences, São Paulo State University, 18618–970, Botucatu, São Paulo, Brazil; Xavier Bichat Medical School, INSERM-CNRS - Université Paris Diderot, FRANCE

## Abstract

Preeclampsia (PE) is considered the leading cause of maternal and perinatal morbidity and mortality. The placenta seems to play an essential role in this disease, probably due to factors involved in its formation and development. The present study aimed to investigate the association between placental lesions, cytokines and angiogenic factors in pregnant women with preeclampsia (PE). We evaluated 20 normotensive pregnant women, 40 with early-onset PE and 80 with late-onset PE. Placental samples were analyzed for histopathology, immunohistochemistry and determination of granulocyte-macrophage colony-stimulating factor (GM-CSF), interleukin-10 (IL-10), transforming growth factor-beta 1 (TGF-β_1_), tumor necrosis factor-alpha (TNF-α), placental growth factor (PlGF), vascular endothelial growth factor (VEGF), fms-like tyrosine-kinase-1 (Flt-1) and endoglin (Eng) levels. Higher percentages of increased syncytial knots and increased perivillous fibrin deposits, and greater levels of TNF-α, TGF-β1and Flt-1 were detected in placentas from early-onset PE. Levels of IL-10, VEGF and PlGF were decreased in PE versus normotensive placentas. Both the TNF-α/IL-10 and sFlt-1/PlGF ratios were higher in placental homogenate of early-onset PE than late-onset PE and control groups. The more severe lesions and the imbalance between TNF-α/IL-10 and PlGF/sFlt-1 in placentas from early-onset PE allows differentiation of early and late-onset PE and suggests higher placental impairment in early-onset PE.

## Introduction

Preeclampsia (PE) is a syndrome that affects 2% to 8% of human pregnancies and constitutes a major cause of maternal and perinatal morbidity and mortality [1]. It is a systemic disease characterized by an inflammatory response and endothelial disorder, and is clinically identified by a combination of hypertension and proteinuria, present after 20 weeks of gestation in a previously normotensive pregnant woman [2,3].

Classically, PE is defined as mild or severe, according to clinical and laboratory parameters and the presence of maternal and fetal complications [3]. Another definition is based on the gestational age at initiation of signs and symptoms and classifies PE into early-onset (starting before 34 gestational weeks) or late-onset (starting at or after 34 gestational weeks) disease [4]. According to this classification, early-onset PE is associated with abnormal uteroplacental perfusion, greater prevalence of placental lesions and neonates with low birth weight [5–7]. These cases are also called placental PE and are frequently associated with poor maternal and neonatal outcomes. Late-onset PE occurs more frequently in patients with underlying chronic inflammatory conditions, and is called maternal PE with lower rates of fetal compromise [7,8].

During normal placentation, the maternal spiral arteries are invaded by extravillous trophoblast cells, resulting in remodeling of these vessels into uteroplacental vessels which ensure adequate blood supply to the placenta and fetus. However, in women with PE the trophoblastic invasion is inadequate and leads to impaired spiral artery remodeling with consequent poor uteroplacental perfusion [9]. These alterations are responsible for placental hypoxia/reperfusion lesions that lead to oxidative stress, a local inflammatory response, production of placental debris and anti-angiogenic factors. These products are disseminated into the maternal circulation and are responsible for the systemic inflammatory response and endothelial dysfunction [2]. Thus, the placenta seems to play a fundamental role in the development of PE, and only its delivery can lead to complete regression of symptoms [10].

Regarding the changes found in placentas of preeclamptic women, most of them are associated with uteroplacental underperfusion such as infarction, and increased fibrin deposits [8,11,12]. Additionally, increased syncytial knots is also reported in preeclamptic placentas [10,13]. Syncytial knots are defined as aggregates of syncytiotrophoblast nuclei located on the surface of terminal villi [14], and they are reported to have variable functions but this has not been clearly defined.

The ischemia and hypoxia resulting from the inappropriate trophoblast invasion lead to an increased production of pro-inflammatory cytokines in the placenta [15]. Tumor necrosis factor alpha (TNF-α) and interleukin-1 (IL-1) are overexpressed and secreted in placentas of preeclamptic women likely due to hypoxia-reoxygenation caused by intermittent placental perfusion [16].

Granulocyte-macrophage colony-stimulating factor (GM-CSF) is a hematopoietic cytokine and potent mediator of cell proliferation and differentiation. However, at high concentrations GM-CSF contributes to the effects of placental hypoxia, which is crucial for the development of PE [17]. Increased production of GM-CSF by cultured decidual cells is induced by TNF-αand IL-1, suggesting that GM-CSF plays a role in macrophage and dendritic cell activation in PE [15]. However, its role in placental villi expression remains unknown.

Low placental production of the anti-inflammatory cytokine IL-10 has been described in PE [18,19]. As IL-10 has strong suppressor activity on pro-inflammatory cytokines TNF-α and interferon-gamma (IFN-γ) it is suggested that placental hypoxia leads to insufficient IL-10 production, resulting in increased or uncontrolled production of pro-inflammatory cytokines [15].

The human placenta is a source of angiogenic molecules, which play an important role in blood vessel formation at the maternal-fetal interface [20]. Current studies suggest that an imbalance in placental production and release of pro- and anti-angiogenic factors contributes to the systemic endothelial cell dysfunction in PE [21]. Therefore, significant reduction of the angiogenic factors placental growth factor (PlGF) and vascular endothelial growth factor (VEGF) as well as increased production of the anti-angiogenic factors soluble endoglin (sEng) and soluble fms-like tyrosine kinase-1 (sFlt-1) have been associated with pathogenesis of PE [22].

Placental growth factor and VEGF are glycoproteins involved in angiogenesis and vasculogenesis, and their reduction can result in poor vascularization and impairment of vascular development during trophoblastic invasion [23,24]. Soluble Flt-1 (sFlt-1) is a soluble form of VEGF receptor that binds to PlGF in the systemic circulation, avoiding the homeostatic PlGF effect on the endothelial cells [22]. Furthermore, high levels of sFlt-1 and reduced levels of PlGF have been correlated with severity of PE [25,26]. Endoglin (Eng), or CD105, is a cell surface co-receptor for TGF-β family members (TGF-β1 and TGF-β3). The soluble form of Endoglin (sEng) is elevated in sera of preeclamptic women, and leads to dysregulated TGF-β signaling in the vasculature. The interaction of TGF-β1 with sEng may damage the binding of TGF-β1 to the endothelial receptors, decreasing endothelial vasodilation activated by nitric oxide synthase [27]. Thus, these anti-angiogenic factors antagonize the effects of pro-angiogenic factors VEGF, PlGF and TGF-β, which are important in the maintenance of the vascular endothelium [20].

Considering the involvement of the cytokine and angiogenic imbalance reported in the pathogenesis of preeclampsia, this study investigated the expression of pro- and anti-inflammatory cytokines as well as pro- and anti-angiogenic factors in the placental tissues of women with early-and late-onset PE, and their association with the most frequent lesions expressed by placentas of these preeclamptic women.

## Material and Methods

### Study population

Placentas were collected from 140 women with singleton pregnancies who delivered by elective cesarean section at the Obstetric Unit of Botucatu Medical School, Botucatu, SP, Brazil between March 2011 and December 2012. Gestational age of the groups was calculated from the last menstrual period and confirmed by early (<12 weeks gestation) ultrasound examination. Preeclampsia was defined as blood pressure ≥140/90 mmHg evaluated on two occasions 2 h apart after 20 weeks of gestation and proteinuria of ≥300 mg/24 h in women with no previous history of hypertension [3]. Proteinuria was measured by the Technicon RAXT automation system.

Preeclamptic women were classified as early-onset PE or late-onset PE according to whether disease manifestation occurred before or from the 34^th^ week of gestation, respectively [4]. Twenty placentas were collected from normotensive pregnant women (controls) without hypertension-related complications that delivery at term (≥ 37 weeks of gestation), 40 from pregnant women with early-onset PE and 80 from women with late-onset PE. Placentas were collected by elective cesarean section, in the absence of delivery labor, for all groups studied. Exclusion criteria included patients in labor, premature rupture of membranes, illicit drug use, and preexisting medical conditions such as diabetes, chronic hypertension and renal disease. Intrauterine growth restriction (IUGR) was defined as a newborn that was at or below the tenth percentile in weight for its gestational age according to the perinatal unit’s criteria at the Botucatu Medical School. The study was approved by the Ethics Committee of the Botucatu Medical School, and the written informed consent was obtained from all women involved in the study. (Protocol number 278/11). For pregnant women with age below 18 years old the written informed consent was obtained from their parents or guardians.

### Sample collection and preparation

Immediately after delivery by cesarean section, placentas were macroscopically examined according to previous guidelines [28]. Placental tissues were obtained by cutting a vertical plane through the full thickness of a central and apparently normal area, including both the fetal and maternal surfaces. Tissues with calcification or clots were avoided. Samples of approximately 2 g of placental tissue were taken and used for histopathologic analysis. For homogenate preparation and immunohistochemical analysis, placental fragments from early-onset PE and late-onset PE women (n = 20 for each group) were randomly selected. Placental fragments from 20 normotensive pregnant women were employed as controls.

### Histopathologic analysis of the placentas

The placental segments were placed in sterile 10% formalin phosphate buffer for 48 h, followed by water washing process for 24 h. After dehydration in alcohol and diaphanization in xylene, placental fragments were embedded in paraffin. Histologic sections of 4 μm thick were stained with hematoxylin and eosin (HE), and analyzed by light microscopy (Nikon Eclipse 50i, Nikon, Tokyo, Japan), at 200X magnification. Microscopic examination was performed without knowledge about group identification or, patient clinical information. The parameters evaluated were the presence of increased syncytial knots, infarction, increased perivillous fibrin deposits and accelerated villous maturation. Syncytial knots were defined as aggregates of five or more syncytiotrophoblastic nuclei protruding from the villous surface that were not in contact with other adjacent villi [14]. The percentage of villi containing syncytial knots was determined by counting villi with one or more knots out of 100 villi in the histologic section stained with HE at 200X magnification. Villous infarction was defined as an ischemic necrosis area of the villi, classified as either present or absent in the histologic sections. Accelerated villous maturation was identified when the villous area did not correlate with the actual gestational week but were smaller and more mature appearance than expected [12]. Increased perivillous fibrin deposits were identified by the presence of fibrin accumulation around the villi and were considered increased when more than one quarter of the villi were totally or partially involved [29].

### Immunohistochemical analysis of placental tissues

The expression of pro-inflammatory and anti-inflammatory cytokines (GM-CSF, TNF-α, IL-10, TGF-β_1_) and angiogenic factors (PlGF, VEGF, Flt-1, Eng) were evaluated. Placental tissues embedded in paraffin were sectioned into 4 μm thick slices and placed on histologic slides, pretreated with Vectabond (Vector Laboratories Inc., Burlingame, CA, USA). Deparaffinization, rehydration and antigen recovery of the material was obtained using Trilogy buffer (Cell Marque Co, Rocklin, CA, USA) in a pressure cooker (Cell Marque) for 15 min. Then, sections were washed with phosphate buffered saline (PBS, pH 7.2), and treated with Peroxide Block (Cell Marque) for 10 min to block endogenous peroxidase and then washed by successive baths in distilled water and PBS. The background was blocked using Background Block (Cell Marque) for 10 min followed by washes in distilled water and PBS. After blocking, sections were incubated for 60 min at 37°C with antigen-specific primary anti-human antibodies. The concentrations of the antibodies were previously standardized using normal and preeclamptic placentas. The following antibodies with respective dilutions were used: murine monoclonal anti-TNF-α [1/600], anti-VEGF [2.5 μg/mL], anti-Eng [1/2000] and anti-Flt-1 [1.25 μg/mL] (Abcam Inc., Cambridge, MA, USA), rabbit polyclonal anti-PlGF [1/50], anti-TGF-β1 [1/1000] and anti-IL-10 [1/800] (Abcam Inc., Cambridge, MA, USA) and murine monoclonal anti-GM-CSF [1/2000] (Thermo Scientific, Rockford, IL, USA) diluted in Antibody Diluent (Cell Marque). After incubation, sections were washed in PBS and subjected to the action of signal Amplifier for murine and rabbit antibodies (Cell Marque) for 10 min at 37°C. A Polymer Detector for murine and rabbit antibodies (Cell Marque) was used for detection of primary antibody with incubation for 10 min at 37°C. After this step sections were washed in PBS and incubated for 5 min in revealing solution containing 10 mg of diaminobenzidine (DAB), 0.2% of hydrogen peroxide (H2O2) and Trizma base -20mm, 1N HCl (Sigma). Sections were counterstained with Harris hematoxylin (Cell Marque) for 20 sec, and then bathed in running water. Dehydration was performed in absolute ethanol sequential baths, alcohol 90%, 80% and 70%, cleared in xylene (four baths) and mounted with coverslips containing Permount (Fisher Scientific, Fair Lawn, NJ, USA).

To conduct the negative control reaction, primary antibody was replaced with a mouse or rabbit serum negative control (Cell Marque) containing the immunoglobulin isotype similar to the primary antibody used.

The expression of cytokines was identified in placental sections by an optical microscope (Olympus CX-31) with 10X ocular and 10, 20 and 40X objectives. Five random fields were photographed in every section of placenta with a 20x objective, and were analyzed by employing the software Image J. The quantification of cytokines and angiogenic factors was obtained in pixels/μm/area.

### Preparation of placental homogenates

To prepare placental homogenates, placental tissues were washed four times in ice-cold PBS to remove remaining blood. After this, 2 g of tissue was placed into a homogenate plastic tube containing 10 mL of ice-cold PBS and protease inhibitors (complete protease inhibitors cocktail; Sigma-Aldrich, St. Louis, MO, USA). The tissue was fully homogenized with a Powergen homogenizer (Fisher Scientific, Pittsburg, PA, USA) for 30 seconds on ice. Homogenates were centrifuged at 12000 *g* for 20 min at 4°C. The supernatant was collected, filtered through a 0.22 μm Millipore membrane, and aliquots were stored at –80°C, until required for cytokine and angiogenic factor determination.

### ELISA quantification

Concentrations of IL-10, TNF-α, TGF-β_1_, GM-CSF, VEGF, PlGF, sEng and sFlt-1 in placental homogenates were determined by enzyme-linked immunosorbent assay (ELISA), using Quantikine ELISA kits (R&D Systems, Minneapolis, MN, USA) according to the manufacturer’s instructions. Results were expressed as pg/g of placental tissue.

### Statistical analysis

Statistical analyses for age, gestational age, blood pressure, proteinuria, and birth weight, as well as cytokine and angiogenic factor concentrations, were performed employing the Kruskal-Wallis nonparametric test. Percentages of placental alterations and IUGR were analyzed employing the chi-square test. Percentages of villi containing syncytial knots were compared among groups by analysis of variance (ANOVA) with the Tukey-Kramer multiple comparisons test. Analyses of association were performed using the Spearman rank correlation test. All statistical analyses were performed using Prisma Statistical software 9 version 5.0 (GraphPad San Diego, Calif., U.S.A.). Differences were considered statistically significant at *P* < 0.05.

## Results

### Subject characteristics

Subject characteristics and perinatal outcomes of all cases enrolled in this study are shown in Table 1. Maternal age was similar in the three groups evaluated. The gestational age at delivery and birth weight of newborns were significantly lower in women with early-onset PE than in those with late-onset PE and controls. Blood pressure values, proteinuria and incidence of IUGR were significantly higher in early-onset PE than in late-onset PE. None of the normotensive pregnant women (controls) had IUGR.

**Table 1:** Subject characteristics and perinatal outcomes.

Characteristics	Normotensive Pregnant women (n = 20)	Early-onset PE (n = 40)	Late-onset PE (n = 80)
**Age (years)**	25 (18–40)	24 (13–36)	25 (14–45)
**Gestational age (weeks)**	38 (37–40)	31 * ^#^ (27–33)	37 (34–42)
**Systolic blood pressure (mmHg)**	110 (103–112)	165 * ^#^ (140–220)	150 * (130–170)
**Diastolic blood pressure (mmHg)**	67 (63–70)	110 * ^#^ (95–140)	100 * (90–125)
**Proteinuria (mg/24 hours)**	< 300	3,960 * ^#^ (320–22,520)	720 * (300–9,520)
**Birthweight (g)**	2,990 (2,890–3,910)	1,283 * ^#^ (535–3,170)	2,955 (1,790–3,805)
**IUGR (%)**	-	48.3 ^+^	11.3

The values of maternal age, gestational age, systolic and diastolic blood pressure and proteinuria are expressed as medians and range; Abbreviations: PE = preeclampsia; IUGR = intrauterine growth restriction

* (p<0.05) *vs* normotensive pregnant women;

^#^ (p<0.01) *vs* late-onset PE (Kruskal-Wallis test);

^+^ (p < 0.01) *vs* late-onset PE (chi-square test)

### Histopathologic results

**Fig 1:**
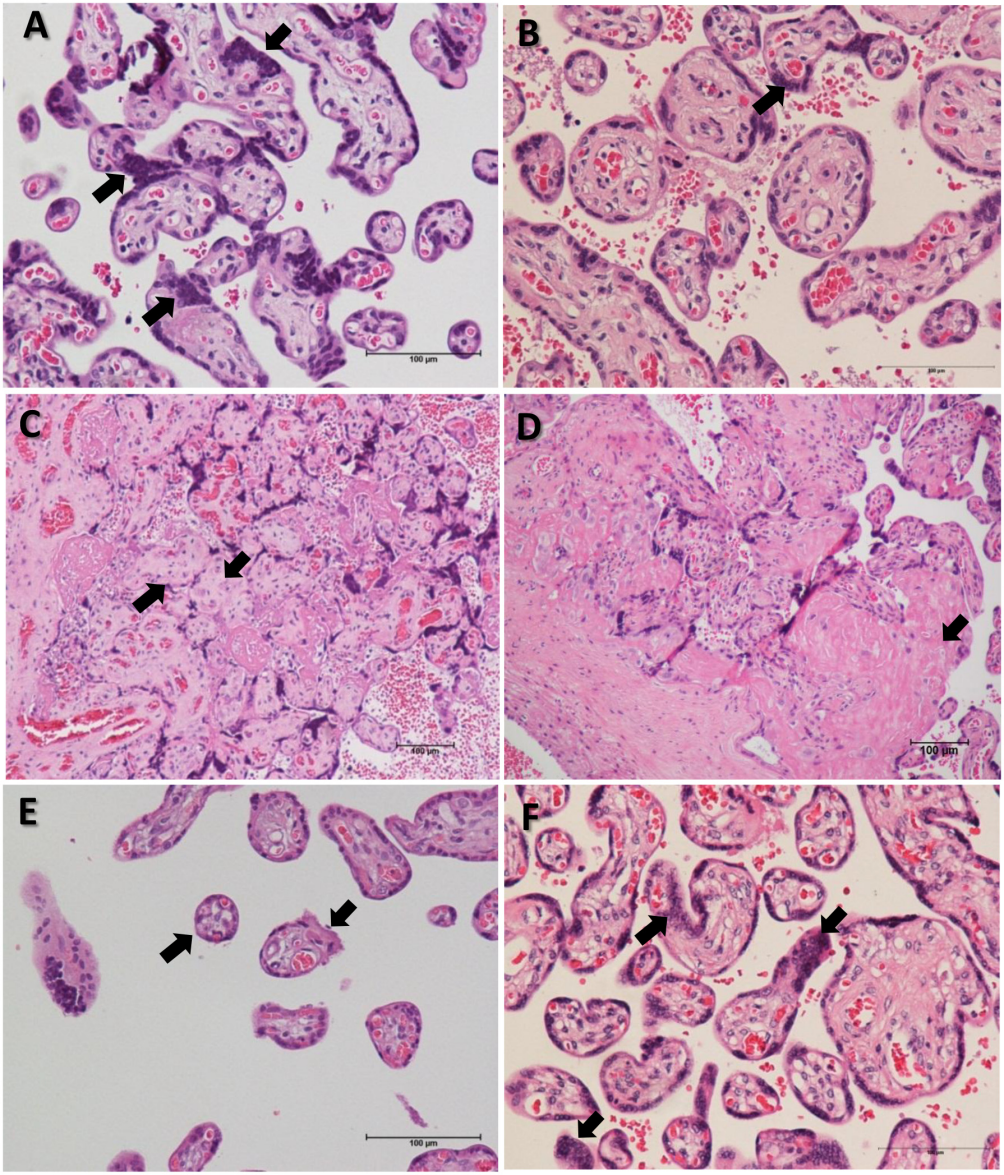
Representative photomicrography of placental histopathology of normotensive pregnant women and women with PE. A) Increase in syncytial knots (arrows) in placenta of a woman with early-onset PE. B) Few syncytial knots (arrow) in placenta of woman with late-onset PE. C) Placental infarction (arrows) of a woman with early-onset PE. D) Increase of fibrin deposits (arrow) in placenta of woman with early-onset PE. E) Accelerated villous maturation (arrows) in the placenta of woman with late-onset PE. F) Presence of syncytial knots (arrows) in the placenta of a normotensive pregnant woman at 38 weeks gestation.

The histopathologic lesions identified in this study were increased syncytial knots, infarction, increased perivillous fibrin deposits and accelerated villous maturation (Fig 1). The percentage of villi containing syncytial knots was higher in placentas from early-onset PE at gestational ages from 24 to 33 weeks (29.4 ± 5.1) compared with late-onset PE at 34 to 36 gestational weeks (19.3 ± 4.5). However, this higher percentage of syncytial knots was similar to those identified in late-onset PE at ≥ 37 gestational weeks (27.3 ± 2.6) or term controls (31.1 ± 2.4).

The percentages of pregnant women with increased perivillous fibrin deposition was significantly more pronounced in early-onset PE (30.0%) compared with late-onset PE (1.3%). No differences between both preeclamptic groups were observed regarding infarction and accelerated villous maturation. The control group expressed less than 1% of placental alterations.

### Cytokine and angiogenic factor expression in placental tissues

**Fig 2:**
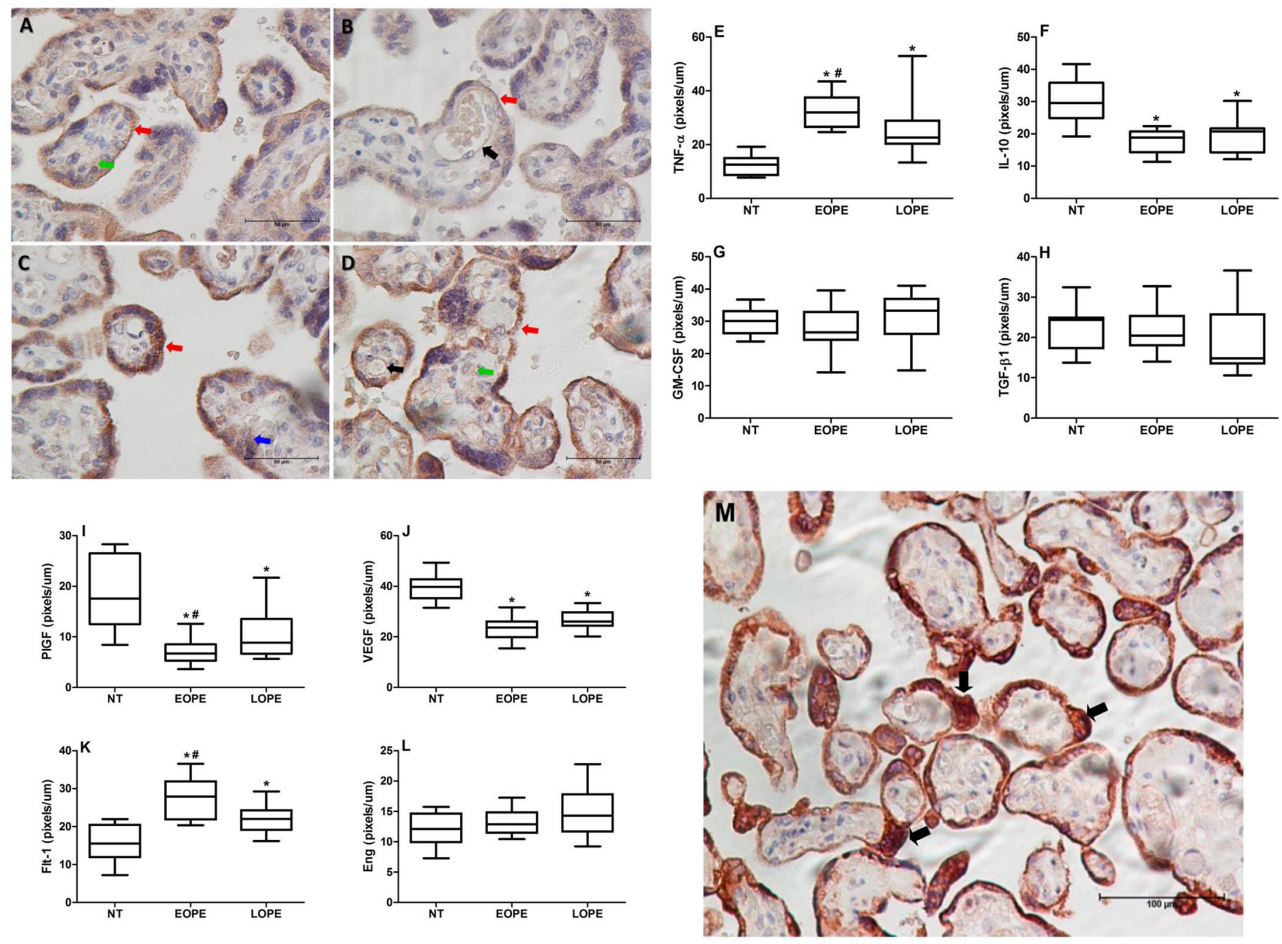
Representative photomicrography showing positivity of cytokine and angiogenic factor in placental sections of pregnant women with PE. A) IL-10; B) PlGF; C) TNF-α; D) Flt-1. Red arrow shows positivity in the syncytiotrophoblast, green arrow shows positivity in cytoplasm of mesenchymal cells, black arrow shows positivity in the fetal capillary endothelium, and blue arrow shows positivity in the cytotrophoblast. Quantitative analysis of the cytokines TNF-α (E), IL-10 (F), GM-CSF (G), TGF-β1 (H), and angiogenic factors PlGF (I), VEGF (J), Flt-1 (K) and Eng (L) expression by placental tissues from normotensive (NT) pregnant women, women with early-onset preeclampsia (EOPE) or late-onset preeclampsia (LOPE). Results are represented in pixels/μm. * (p <0.05) *vs* normotensive group; # (p < 0.05) *vs* late-onset PE. Higher intensity of Flt-1 (arrows) expressed in syncytial knots in placenta of an early-onset preeclamptic women at 31 weeks of gestation (M).

Fig 2 is representative of the cytokines and angiogenic factors expressed by placental tissues of preeclamptic women. According to our analysis, TNF-α, GM-CSF, IL-10 and TGF-β1 were expressed by syncytiotrophoblast cells. Tumor necrosis factor-alpha and GM-CSF were also expressed by cytotrophoblast cells, whereas IL-10 was expressed by mesenchymal cells. Transforming growth factor-beta 1 was also expressed by endothelial cells of the fetal capillaries.

The pro-angiogenic factors VEGF and PlGF were detected in syncytiotrophoblast and endothelial cells of the fetal capillaries. Vascular endothelial growth factor was also expressed by mesenchymal cells, and Flt-1 expression occurred in the same cells that expressed VEGF. Endoglin was expressed only by syncytiotrophoblast cells.

All cytokines and angiogenic factors evaluated showed the same localization in the three groups studied. However, differences in terms of intensity of expression were found, and were quantified using the software Image J (Fig 2E–2L). The intensity of TNF-αexpression was significantly higher in placentas of preeclamptic groups than in placentas of the normotensive group (Fig 2E). Interestingly, the expression of TNF-α by placentas of the early-onset PE group was also significantly higher compared to the late-onset PE group. The expression of IL-10 was significantly lower in both groups of preeclamptic placentas than in normotensive placentas (Fig 2F). No differences were found regarding GM-CSF or TGF-β1 expression in the three groups studied (Fig 2G and 2H). Analysis of PlGF and VEGF showed that both pro-angiogenic factors were significantly decreased in preeclamptic placentas than in normotensive ones (Fig 2I and 2J). In the early-onset PE group, PlGF expression was also significantly lower than in the late-onset PE group. The expression of Flt-1 was higher in preeclamptic placentas than in normotensive placentas and was significantly more intense in the early-onset PE group (Fig 2K). The expression of Eng was not different in any group evaluated (Fig 2L). High positivity of Flt-1 was detected in syncytial knots of placental villi of early-onset PE group (Fig 2M).

### Cytokines and angiogenic factors in placental homogenates

The concentrations of TNF-α, TGF-β1, sFlt-1 and Eng in placental homogenates were significantly higher in the early-onset PE group compared with the late-onset PE and normotensive groups (Table 2). The levels of IL-10, PlGF and VEGF were lower in placentas of both preeclamptic groups than in the normotensive group. Placental growth factor was also lower in the early-onset than the late-onset PE group. No significant differences were detected regarding GM-CSF levels among the groups studied. Association analysis between sFlt-1 levels in placental homogenates and percentage of syncytial knots expression showed a moderate positive correlation in both early-onset PE (r = 0.6737; p = 0.0011) and late-onset PE (r = 0.5017; p = 0.0242). No correlation between these parameters (r = 0.2903; p = 0.2144) was observed in the normotensive pregnant group. No significative correlation between PlGF and syncytial knots were detected in early-onset PE (r = 0.2096; p = 0.3751), late-onset PE (r = 0.3704; p = 0.1079) and normotensive (r = 0.3378; p = 0.1453) groups.

**Table 2:** Concentrations of TNF-α, IL-10, TGF-β1, GM-CSF, VEGF, PlGF, sFlt-1 and sEng in placental homogenates from pregnant women with early-onset PE and late-onset PE and normotensive pregnant women.

Parameters (pg/g of tissue)	Early-onset PE	Late-onset PE	Normotensive
**TNF-α**	96.28 *^#^(24.03–173.38)	49.50 * (16.98–96.34)	28.96 (7.07–80.74)
**IL-10**	6.21* (3.48–8.34)	8.36 * (4.83–12.46)	15.31 (7.76–29.67)
**TGF-β1**	474.01* ^#^ (207.10–1330.95)	312.04 (212.05–647.48)	324.79 (121.68–814.61)
**GM-CSF**	65.57 (25.20–113.77)	44.20 (30.47–93.04)	54.90 (24.73–103.77)
**PlGF**	40.79 * ^#^ (13.28–99.80)	77.28 * (37.83–147.34)	120.55 (48.40–243.57)
**VEGF**	16.98 * (4.73–49.12)	13.73 * (6.45–35.01)	32.19 (19.23–114.25)
**sFlt-1**	4035.22 * ^#^(2593.05–8339.62)	2327.66 (1509.15–5629.44)	2847.73 (1483.82–3950.97)
**sEng**	49.35 * ^#^ (35.19–132.36)	27.06 (18.64–64.70)	35.63 (15.63–83.78)

Results are expressed as median values and (range). Abbreviations: PE = preeclampsia.

* (p<0.05) *vs* normotensive group;

^**#**^ (p <0.05) *vs* late-onset PE group.

**Fig 3:**
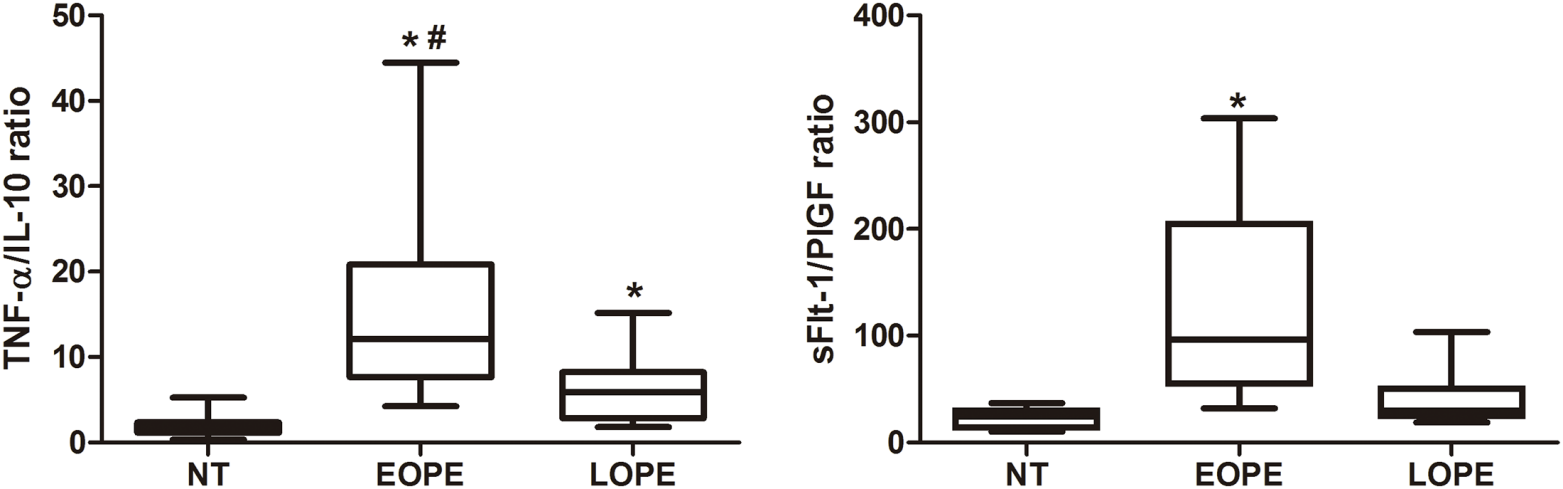
TNF-α/IL-10 ratio (A) and sFlt-1/PlGF ratio (B) determined in placental homogenates from normotensive (NT) pregnant women, women with early-onset preeclampsia (EOPE) and late-onset preeclampsia (LOPE). * (p <0.05) *vs* normotensive group; **#** (p <0.05) *vs* late-onset PE group.

The results of the TNF-α/IL-10 ratio as well as the sFlt-1/PlGF ratio determined in placental homogenates from normotensive pregnant women and women with early-onset PE and late-onset PE are also represented (Fig 3A and 3B). Median values of the TNF-α/IL-10 ratio (25^th^– 75^th^ percentile) were significantly higher in homogenates of early-onset PE (15.38; 5.08–36.44) than late-onset PE (5.88; 2.03–13.42) and controls (1.66; 1.05–4.74). Statistical differences were also detected between the late-onset PE and normotensive groups. In the early-onset PE group, median values of the sFlt-1/PlGF ratio (25^th^– 75^th^ percentile) (113.55; 44.04–205.86) were significantly higher than in the late-onset PE (29.7; 20.46–66.07) and normotensive (24.49; 11.12–30.87) groups.

## Discussion

Although the etiopathology of PE is still not clearly defined, there is consistent evidence that placental alterations constitute the main factors responsible for the development of the disease and its severity [2,12]. This study investigated the most frequent lesions expressed by placentas of women with early-onset and late-onset PE. The expression of pro- and anti-inflammatory cytokines as well as pro- and anti-angiogenic factors was analyzed in placental tissue and their levels in placental homogenates were also evaluated to better understand their involvement in the pathophysiology of PE.

Regarding placental lesions, an increased prevalence of increased syncytial knots and increased perivillous fibrin deposits were present in pregnant women with early-onset PE (24–33 weeks of gestation) than late-onset PE (34–36 weeks). Our data are consistent with those reported by Kovo and collaborators [7]. High percentages of syncytial knots are rarely seen in immature placental villi [14]. When present in preterm pregnancies with hypertension, increased syncytial knots shows a positive correlation with the severity of the disease and fetal growth restriction [30]. It had may also been interpreted as syncytial hyperplasia occurring in response to trophoblastic ischemia or hypoxia [31]. In this study, patients with early-onset PE showed more severe conditions, with higher levels of proteinuria and blood pressure and a higher incidence of IUGR. Interestingly, women with late-onset PE diagnosed after 37 weeks also showed a significantly higher percentage of increased syncytial knots than those patients with late-onset PE at 34 to 36 gestational weeks, similar to the placenta of normotensive pregnant women at term. These findings suggest that the percentage of syncytial knots in placentas of women with late-onset PE after 37 weeks may be related to normal villous maturation as observed in normotensive pregnant women.

The percentage of increased fibrin deposits was significantly higher in placentas from early-onset PE than late-onset PE. The excessive fibrin deposition in conjunction with the hypoxia-associated apoptosis/necrosis of syncytiotrophoblasts may play a role in the severe outcomes and maternal symptoms in PE [32]. The increased syncytial knots and fibrin deposits in placentas from early-onset PE suggest that this clinical presentation fits more with placental preeclampsia than maternal preeclampsia, according to the new definitions by Redman [33]. Both early-onset and late-onset PE groups showed increased percentages of placental infarction and accelerated villous maturation when compared to normal term placentas. No significant differences were found between the two PE groups. In contrast, van der Merwe and collaborators [5] observed that placental infarction is more frequent in patients with early-onset PE than late-onset PE. Another point of view was presented by Vinnars and collaborators [12] who reported that placental infarctions in PE are related to the severity of the disease, regardless of gestational age.

To better understand the association of placental lesions with expression of cytokines and angiogenic factors in placental tissues of preeclamptic pregnant women we evaluated the expression of pro- and anti-inflammatory cytokines as well as pro- and anti-angiogenic factors by immunohistochemistry, and the levels of these factors in placental homogenates by enzyme immunoassay. All cytokines (TNF-α, GM-CSF, IL-10 and TGF-β1) and angiogenic factors (PlGF, VEGF, Flt-1 and Eng) were detected in the syncytiotrophoblast. Expression of TNF-α and GM-CSF was also observed in the cytoplasm of cytotrophoblast cells. Interleukin-10, VEGF and Flt-1 were present in the cytoplasm of mesenchymal cells, whereas TGF-β1, VEGF and Flt-1 were expressed in the endothelium of fetal capillaries. These locations of cytokines and angiogenic factors in placental structures were similar among the groups of normotensive and preeclamptic patients. However, the difference between groups was in the degree of intensity of labeling of these cytokines and angiogenic factors in placental tissue. Although TNF-α was expressed equally in the syncytiotrophoblast and cytotrophoblast of placental villi in the three groups studied, the intensity of TNF-α labeling was significantly higher in placentas of the early-onset PE group than the late-onset PE and normotensive groups. The TNF-α concentration in placental homogenates agreed with the cytokine expression in the tissue, with higher levels in the early-onset PE group than the late-onset PE and normotensive groups. These results are in agreement with other studies in the literature, showing higher expression of TNF-α in the syncytiotrophoblast, cytotrophoblast, vascular endothelial cells, decidual cells and villous stroma associated with PE severity [34,35].

Unlike the high expression of TNF-α in preeclamptic patients, IL-10 showed significantly less expression in placental tissue and significantly lower levels in placental homogenates obtained from PE patients compared with the normotensive group. According to the literature, pregnant women with PE have deficient production of IL-10 in the placenta at term and a lower concentration in the circulation or show less endogenous production by monocytes in peripheral blood [18,19,36,37].

The expression of TGF-β1 and GM-CSF showed no significant differences between groups by immunohistochemistry, but higher levels of TGF-β1 were detected in placental homogenates from the early-onset PE group. In the literature, high expression of TGF-β1 is reported in the placental villous syncytiotrophoblast of preeclamptic women, without differences between mild and severe cases of the disease. This cytokine may participate in vascular pathogenic process of endothelial damage of PE [38].

The angiogenesis and vasculogenesis processes are dependent on significant production of endothelial growth factors. An adequate balance between the angiogenic factors VEGF and PlGF with their specific receptors is important for the effective vasculogenesis, angiogenesis and placental development during pregnancy [39]. In this study, we observed that both VEGF and PlGF showed significantly decreased expression in the syncytiotrophoblast and endothelium from fetal capillaries in the placenta of pregnant women with PE compared to the normotensive pregnant group. These results suggest the occurrence of abnormalities in the growth and formation of new vessels and hence an anomaly in placental vascular remodeling, especially in pregnant women with early-onset PE that had lower intensity of PlGF expression compared to pregnant women with late-onset PE. The lower expression of PlGF and VEGF in placental tissues of pregnant women with PE may be associated with the pathogenesis of the disease [40,41].

Our results showed more intense labeling of Flt-1 in placental tissues of pregnant women with PE than in normotensive pregnant women, and higher concentrations of sFlt-1 in placental homogenates in early-onset PE, in agreement with other authors [39,42]. A possible explanation for the increase in Flt-1 seen in preeclamptic placentas, particularly in early-onset preeclampsia may be related to the early events in gestation that occur in preeclampsia. Placental ischemia caused by insufficient maternal spiral arterial remodeling led to increase in hypoxia-inducible factor (HIF-1), a transcription factor that acts on target genes, including the anti-angiogenic factor sFlt-1 which is thus regulated by oxygen tension in placenta [43]. According to Tripathi et al [39] it is possible that an ischemic placenta could induce expression of membranous VEGFR-1 and release of sVEGFR-1 from villous trophoblast cells, which are in direct contact with maternal blood in the intervillous space. Thus, it seems that in preeclamptic patients the higher expression of Flt-1 may generate more sFlt-1 that is associated with the severity of PE. During pregnancy, the placenta is the main source of sFlt-1 in maternal circulation [25], with greater production in placentas of pregnant women with PE compared to normal pregnant women [42]. It is known that altered placentas release greater amounts of microparticles, debris and anti-angiogenic factors into the maternal circulation. Soluble fms-like tyrosine-kinase-1 is released in higher amounts by preeclamptic placentas and it has been implicated in the endothelial dysfunction observed in the disease [44]. Other studies showed that circulating levels of sFlt-1 are increased in pregnant women with PE, especially in severe PE and early-onset PE [27,45]. Thus, the high sensitivity and specificity of the soluble VEGF Receptor-1 or sFlt-1 concentrations in the plasma of pregnant women with PE may suggest the use of their diagnostic utility as a sensitive biomarker for early-onset PE [39].

The consequences of a higher circulatory sFlt-1/PlGF ratio have been described to be more intense among women with early-onset PE than in those with late-onset PE [26]. Recently, Baltajian and collaborators [46] demonstrated that preeclamptic patients with a higher circulating sFlt-1/PlGF ratio have higher rates of placental vascular lesions, and they suggested that altered placental tissue is the main source of this anti-angiogenic factor. Our work is the first one to show a higher sFlt-1/PlGF ratio in placental homogenates of early-onset PE, which is associated with lesion severity. The higher percentage of syncytial knots identified in this study was correlated with the higher levels of sFlt-1 in placental homogenates from both early-onset and late-onset PE groups, with the largest correlation coefficient detected in early-onset PE. Also, greater intensity of Flt-1 labeling by immunohistochemistry was detected in syncytial knots of placental villi in the early-onset PE group. It has been reported that placentas from preeclamptic women have more syncytial knots that are loaded with Flt-1 protein compared to those from normal pregnancies [39]. The detachment of syncytial knots from these placentas result in free syncytial aggregates that represent autonomous sources of sFlt-1 delivery into maternal circulation [47]. Therefore, we suppose that the higher levels of sFlt-1 detected in placental homogenates of preeclamptic placentas may be a result of sFlt-1 release by syncytial knots.

Together, the results of this study showed that placentas of pregnant women with PE have an imbalance between TNF-α and IL-10 and between PlGF and Flt-1 and sFlt-1, as detected by the expression of these factors in placental villi as well as in placental homogenates. The higher intensity of TNF-α expression and lower PlGF expression in the placenta of pregnant women with early-onset PE allows differentiation of these two forms of the disease and suggests greater placental impairment in early-onset PE. These data corroborate the theory that early-onset PE has placental alteration as its main pathologic factor.
